# Randomised Double-Blind Trial of Combination Antibiotic Therapy in Rheumatoid Arthritis

**DOI:** 10.1155/2011/585497

**Published:** 2011-07-06

**Authors:** Angela Smith, Caroline Doré, Peter Charles, Alena Vallance, Tara Potier, Charles Mackworth-Young

**Affiliations:** ^1^Kennedy Institute of Rheumatology, 1 Aspenlea Road, London W6 8LH, UK; ^2^MRC Clinical Trials Unit, 222 Euston Road, London NW1 2DA, UK; ^3^Charing Cross Hospital, Fulham Palace Road, London W6 8RF, UK

## Abstract

* Objective*. A combination of intravenous clindamycin and oral tetracycline has been used for many years as a treatment for active rheumatoid arthritis (RA), despite the absence of good evidence for its efficacy. A single-blind pilot study of this therapy suggested that a double-blind placebo-controlled trial was warranted. *Methods*. Patients with active RA were randomised in a 2 : 1 ratio to receive active treatment or placebo for 25 weeks. The active treatment consisted of intravenous clindamycin in a reducing regime, and oral tetracycline twice daily three times a week. 50 patients were to be recruited. The primary outcome measure was the proportion of patients achieving an ACR20 response. *Results*. An interim statistical analysis was performed after 20 patients had completed the study. Two patients in the active group achieved an ACR20 response, with none in the placebo group (NS). There was a better ESR20 response in the placebo group (*P* = .02). There were no other significant differences between the groups. The results indicated that it was unlikely that a significant difference in ACR20 response would emerge if the remaining 30 patients were recruited. The trial was therefore halted. *Conclusion*. This antibiotic regime is unlikely to be a valuable therapy for active rheumatoid arthritis.

## 1. Introduction

Many drugs are used to treat RA. These include nonsteroidal anti-inflammatory drugs (NSAIDs), “second-line” drugs, corticosteroids and biological agents. All of these therapies have significant drawbacks in terms of efficacy, side effects, or cost [1, 2]. For many patients; therefore, current therapy remains unsatisfactory, and there remains a need to find treatments that are effective, well tolerated, and relatively inexpensive.

There has been growing interest in the use of antibiotics for the treatment of RA, notably tetracyclines [3]. These were initially used by Brown et al. in uncontrolled studies in patients with RA [4]. The original rationale for their use was the hypothesis that RA may be caused by infection with *Mycoplasma* or similar organisms [5]. This now seems unlikely, and subsequent studies have suggested other actions for this class of drug, including the inhibition of matrix metalloproteinases [6]. Three double-blind, placebo-controlled trials of minocycline in active RA have shown significant improvement in most clinical and laboratory measurements in the treatment group compared with the placebo group [7–9]. In the treatment of early RA, minocycline has also been shown to be superior to hydroxychloroquine [10] while doxycycline has been shown to enhance the response to methotrexate [11]. 

On the basis of other bacteria being a possible causative or exacerbating agent in RA [4], a combination of intravenous (IV) clindamycin and oral tetracycline has been used by some physicians for many years as a treatment for active rheumatoid arthritis. However, apart from unpublished, anecdotal reports, there was no controlled evidence for the efficacy of this regime. In view of this, we performed a single-blind, controlled pilot study of this antibiotic combination [12]. Half of the patients were given active treatment in addition to their usual therapy, and half were given no additional treatment. For ethical reasons patients in this latter group were not given a placebo, and all participants knew into which group they had been allocated. The treatment was given for a year, and patients were followed up for a further six months.

Nine out of eleven patients in the treatment group completed the first 12 months of the study, while only three of ten patients in the control group did (*P* < .05). Patients who withdrew did so because of lack of improvement in their condition. Five patients in the treatment group had an ACR20 response at 12 months, while none of the control patients did (*P* < .05). Improvement generally occurred within six months of starting the treatment. Further evidence for the efficacy of the treatment was that it was not well maintained once it had been withdrawn. No significant side effects were encountered.

These results suggested that this combined antibiotic therapy might be useful in the management of active RA although they could have been explained by a placebo response to the therapy. They indicated that a double-blind, placebo-controlled trial of the therapy was justified.

## 2. Patients and Methods

### 2.1. Patients

Male and female patients aged between 18 and 80 years attending the Rheumatology Clinic at Charing Cross Hospital were invited to take part in the study. All had classical RA of at least six months' duration, as defined by the American College of Rheumatology classification criteria [13]. All patients had active synovitis, a disease activity score (DAS28) of greater than 3.8 [14], and radiographic erosions. All patients had “failed” methotrexate or sulphasalazine: either they had had an inadequate response (as defined by persistence of active synovitis) to methotrexate (at least 7.5 mg per week) or sulphasalazine (at least 1,000 mg twice daily) for at least three months or they had been unable to take or tolerate either drug at the above doses. Patients could be taking other second-line antirheumatic drugs (e.g., sulphasalazine, methotrexate, hydroxychloroquine, azathioprine, leflunomide, intramuscular gold, oral gold, or penicillamine), but the dose of any of these had to have been unchanged for at least three months. They could also be on prednisolone, up to a maximum dose of 7.5 mg per day, unchanged for at least three months. Patients could be taking NSAIDs and/or simple analgesics (e.g., paracetamol, coproxamol, or codeine).

Exclusion criteria included severely incapacity (Steinbrocker functional grade IV [15]), chronic/ recurrent infection (e.g., chronic bronchitis, recurrent sinusitis), other infections, immunodeficiency, malignancy, inflammatory bowel disease, other diarrhoeal states, other major illness, history of adverse reactions to tetracycline, clindamycin, or similar antibiotics, and pregnancy or lactation.

After recruitment, patients were randomly assigned in a 2 : 1 ratio to one of two treatment groups: Group I (antibiotic group) and Group II (control group).

### 2.2. Treatment Regime: General

The patients in Group I received IV and oral antibiotics as below. Patients in Group II received normal saline infusions and matched placebo tablets, as detailed below. Apart from the antibiotic therapy, the protocol for treatment (other drugs), assessment, and withdrawal from the study was identical for the two groups of patients. Patients were blinded as to which group they had been randomised, as were all clinicians and assessors.

### 2.3. Trial Drugs

The drug regime was the same as that commonly used in clinical practice (Hornett G, personal communication), and employed in the pilot study [12]. After an initial assessment (see below), patients in Group I started receiving IV infusions according to the regime in Table 1. Each infusion was given slowly in 250 mL normal saline over half an hour. Patients in Group II received IV saline without clindamycin according to the same regime. In addition, patients took oral tetracycline 250 mg (Group I) or placebo (Group II) twice a day three times per week. This regime was continued for each patient for 25 weeks. There were a total of 18 IV infusions for each patient. The maximum time between initial assessment and the first infusion was one month.

### 2.4. Other Drugs

During the study, the doses of second-line agents and prednisolone remained unaltered, if taken. If more active treatment of the rheumatoid arthritis was required, patients were offered joint aspiration and intra-articular injection of steroid (methylprednisolone 40–80 mg or hydrocortisone 12.5–25 mg) into one or more inflamed joints, up to a maximum of three injections. If further steroid was required or it became necessary to use another immunosuppressive drug, the patient left the study, and the event was recorded as a treatment failure. Patients were allowed to continue taking NSAIDs and/or simple analgesics and to alter the doses of these as required.

### 2.5. Withdrawal

Patients were free to withdraw from the study at any time and were withdrawn if this was thought necessary for any other reason by the supervising clinician.

### 2.6. Assessments

Patients were assessed at the outset of the study, and then every two months until the end of the study (25 weeks), that is, four assessments in all. The following were used:

#### 2.6.1. Clinical

American College of Rheumatology (ACR) Core Data Set [16]; this included the following:

- patient's assessment of joint pain (visual analogue scale);
- patient's global assessment of disease activity (visual analogue scale);
- assessor's global assessment of disease activity (visual analogue scale);
- functional assessment completion of health assessment questionnaire (HAQ) by patient;
- number of swollen and number of tender joints (determined by physical examination of 28 joints).

NSAID and analgesic use.

The dose of oral prednisolone, and all other drugs.

Any joints which had been injected with steroid during the study were excluded from the assessment.

#### 2.6.2. Laboratory

Standard blood tests (full blood count, erythrocyte sedimentation rate (ESR), C-reactive protein (CRP), and liver function tests)

Serum matrix metalloproteinase levels (MMP-1 and MMP-3) were measured, using commercially available kits (R&D Systems, Abingdon, UK). Urinary pyridinoline cross-links (PYD) were detected using a specific monoclonal antibody in a commercial kit (Metra, Quidel, San Diego, Calif, USA) [17].

### 2.7. Outcome Measures

The primary outcome measure was the achievement of an ACR 20% response [16] at six months. This was defined as:

- >20% reduction in number of swollen and tender joints;
- >20% improvement in at least three out of five of the following:
  - CRP;
  - patient's assessment of joint pain (visual analogue scale);
  - patient's global assessment of disease activity (visual analogue scale);
  - assessor's global assessment of disease activity (visual analogue scale);
  - functional assessment completion of health assessment questionnaire (HAQ) by the patient.

Other outcome measures included the following:

- individual components of the ACR score, as above;
- withdrawal from the study because of a greater than 20% deterioration according to ACR response;
- number of intraarticular steroid injections given;
- the use of NSAIDs and analgesics.

If withdrawal occurred for any reason, a final clinical and laboratory assessment was performed at this point.

### 2.8. Statistics

#### 2.8.1. Randomization Ratio

 A participant ratio of 2 : 1 between Groups I and II was chosen in order (i) to obtain more information on adverse effects in the treatment group, (ii) to aid recruitment, and (iii) to reduce withdrawals from the study. 

#### 2.8.2. Sample Size Calculations

 The primary outcome measure was the proportion of patients who achieved an ACR 20% response. It was assumed that only 5% of the patients receiving standard therapy would respond. A sample size of 30 patients in Group I (active treatment) and 15 patients in Group II (placebo) would be sufficient to detect a response rate of 50% in the active-treated group using the 5% significance level with a power of 80%. The patient numbers were increased by approximately 10% to take account of individuals dropping out of the study for reasons other than disease progression. This gave 33 patients in Group I and 17 patients in Group II, and, therefore, a total of 50.

#### 2.8.3. Statistical Analysis

 The statistical analysis was restricted to the measurements at baseline and six months. For those patients who withdrew from the study, measurements from the final assessment were used in the statistical analysis.

The proportion of patients who achieved an ACR 20% response (relative to baseline) at six months were compared in the two randomised groups, using Fisher's exact test. Patients who were withdrawn from the study were included in this analysis as nonresponders.

For continuous outcome measures a two-sample *t*-test was used to compare the change from baseline in the two randomised groups.

### 2.9. Ethical Approval

Ethical approval for this project was obtained from the Hammersmith Hospital Research Ethics Committee.

## 3. Results

It had been planned to recruit 50 patients to the trial: 33 patients in Group I (active treatment) and 17 patients in Group II (placebo). After the 20th patient had completed the study, an interim statistical analysis was performed. This analysis showed that in almost all outcome measures, there was no trend towards a difference between active treatment and placebo. The trial was, therefore, halted at this point. The following results are, therefore, those obtained on those 20 patients. 

Twelve patients were randomised to active treatment and 8 to placebo treatment. Assessments were made at weeks 1, 8, 16, and 25. Week 1 was the start of treatment and week 25 the end of treatment. Table 2 summarises the baseline characteristics of each group. The two randomised groups were similar at baseline.

The primary outcome measure was the ACR20 response at the end of treatment (week 25). Improvement was calculated by comparing the value at week 25 with the value at baseline (week 1). Improvements corresponded to a decrease for each of the 7 variables. These comparisons are summarised in Table 3. A total of 15 of the 20 patients completed the 25 weeks of followup. Patients who did not complete the treatment period were considered not to have achieved a 20% improvement. Two patients in the active group achieved an ACR20 response, while none of the patients in the placebo group did (not significant).

All five of the patients who did not complete the trial were on active therapy. Three patients were withdrawn due to disease progression, and two were withdrawn due to protocol transgression. This difference in completion rates was statistically significant (*P* = .06). There was also a significant difference (*P* = .02) in the percentage of patients with a 20% ESR response, with a higher improvement rate in the placebo group.

The sample size calculation assumed that 5% of placebo and 50% of active patients would achieve an ACR20 response, a difference in response rates of 45%. The 95% confidence interval for the (placebo-active) difference in response rates was 38 to 4, which excludes a difference of 45%. The 95% confidence interval for an ACR20 response in the active treatment group was 2 to 48%, which excludes the response rate of 50% used in the sample size calculation. This made it very unlikely that there would be a statistically significant difference in ACR20 response between the two groups if the trial were continued to completion and a total of 50 patients recruited.

An analysis of covariance was performed to compare the two treatments for the assessments at the end of trial, including the baseline value as a covariate. This enabled comparison of the final status of the two groups after allowing for their initial status. The results showed no discernable trend towards a difference between the two groups.

For the whole cohort of 20 patients, there was significant correlation between levels of MMP-1 and HAQ score (*P* = .02), MMP-3 and CRP (*P* = .001), MMP-3 and patient global assessment (*P* = .068), and PYD and HAQ (*P* = .054). There was no significant correlation between MMP-1, MMP-3, and PYD levels, and any of the other measures of disease activity. There was no significant difference in the change in MMP-1, MMP-3, or PYD levels between the two groups.

## 4. Discussion

In this trial we used the same treatment regime—intravenous clindamycin and oral tetracycline—as that employed in the pilot study. Based on our power calculation, we had planned to recruit 50 patients in a 2 : 1 ratio (active to placebo treatment). However, after 20 patients had completed the study, an interim statistical analysis showed that it would be unlikely that a significant difference would emerge between the treatment groups if we were to recruit the remaining 30 patients. The trial was therefore halted.

The results reported here on the first 20 patients did show some differences between the two groups. Two out of the 12 patients (17%) in the active treatment group (Group I) showed an ACR20 response, while none of the patients receiving placebo did (Group II). However, the confidence intervals were wide. If this trend were continued, we would have had to recruit 126 patients in order for the difference to become significant.

Against this trend, the results showed a higher number of withdrawals due to disease progression in Group I compared with Group II. There was a statistically significant greater improvement in ESR in Group II compared with Group I.

There are a number of possibilities to explain the apparent lack of benefit of the active treatment. It is likely that receiving intravenous infusions on a regular basis has a significant placebo effect. This could explain why there was an apparent benefit in the single-blind but not in the double-blind study. It may be that a small number of patients with RA do genuinely respond to the antibiotic therapy, but that our study was underpowered to detect this. If this is the case, it could be argued that the number needed to treat would be too high for this to be a valuable therapy. Another possibility is the difference in oral antibiotic: previous studies in this field have generally used minocycline or doxycycline [9–13], and the dose of tetracycline used in this trial was comparatively low; in earlier studies, minocycline and doxycycline have been given twice daily [9–13].

The literature shows robust evidence for the efficacy of various tetracycline-based antibiotic regimes in modifying the activity of rheumatoid arthritis. The results of the present trial would be consistent with the hypothesis that it is the anti-inflammatory rather than the antibacterial activity of tetracyclines that confers this benefit.

The particular combination of clindamycin and tetracycline used in this trial has been employed extensively in clinical practice. However, the results indicate that it may not be suitable for further study.

## Figures and Tables

**Table 1:** Treatment and assessment regimes for patients in Group I (active treatment). Clindamycin infusions were given in 250 mL normal saline over one hour. Patients in Group II (placebo) received placebo infusions of normal saline and placebo tablets according to the same regime; they had the same assessment regime. bd: taken twice a day (morning and evening). × 3/wk: three times per week. Assessments: C, clinical; L, laboratory.

Time	Clindamycin infusion	Tetracycline	Assessment	
			C	L
Day 1	300 mg	250 mg bd	+	+
Day 2	300 mg	none		
Day 3	600 mg	250 mg bd		
Day 4	600 mg	none		
Day 5	900 mg	250 mg bd		
Weeks 2–4	900 mg per week	250 mg bd × 3/wk		
Weeks 5–8	900 mg fortnightly	250 mg bd × 3/wk		
At week 9			+	+
Weeks 9–16	900 mg fortnightly	250 mg bd × 3/wk		
At week 17			+	+
Weeks 17–24	900 mg fortnightly	250 mg bd × 3/wk		
At week 25			+	+

**Table 2:** Baseline characteristics of each group.

Variable	Placebo (*n* = 8)		Active (*n* = 12)	
	Mean	SD	Mean	SD
Assess	5.0	3.0	4.7	2.0
DAS	4.7	0.9	4.7	0.7
EMS	75	108	154	405
HAQ	1.7	0.4	1.3	0.7
CRP	12	15	23	19
ESR	29	16	26	16
Fatigue	6.6	1.6	6.3	1.5
Pain	4.2	2.7	5.2	1.9
Pt. assess	4.2	2.9	5.4	1.8
Swollen joints	11	4	12	5
Tender joints	6	3	7	3

Assess: physician global assessment, VAS; DAS: DAS disease activity score; EMS: early morning stiffness; HAQ: HAQ disability score; Fatigue: fatigue assessment, VAS; Pain: patient assessment of pain, VAS; Pt. assess: patient global assessment, VAS; Swollen joints: swollen joint count; Tender joints: tender joint count.

**Table 3:** Number and percentage of patients with an ACR20 response, together with the number of patients with a 20% improvement for each core variable. 20 after each variable indicates 20% improvement.

Variable	Placebo (*n* = 8)		Active (*n* = 12)		Placebo-active		*P* value
	Number	%	Number	%	Difference %	95% CI	
ACR20	0	0	2	17	−17	−38 to 4	.5
Completed	8	100	7	58	42	14 to 70	.06
HAQ20	0	0	2	17	−17	−38 to 4	.5
VAS Assess.20	5	63	4	33	29	−14 to 72	.4
Pat. Pain20	1	13	3	25	−13	−46 to 21	.6
Pat. Dis. Assess.20	1	13	2	17	−4	−35 to 27	1.0
CRP20	3	38	4	33	4	−39 to 47	1.0
ESR20	5	63	1	8	54	17 to 91	.02
Swollen joint20	4	50	6	50	0	−45 to 45	1.0
Tender joint20	6	75	6	50	25	−16 to 66	.4
